# Liquid-Mediated Si-OH Healing of ZSM-22 Zeolites for Improved Performance in N-Decane Hydroisomerization

**DOI:** 10.3390/molecules30163319

**Published:** 2025-08-08

**Authors:** Tong Chang, Renhui Deng, Jiaxin Xie, Jianyu Tang, Wenyao Gu, Zimin Peng, Suyao Liu

**Affiliations:** 1 Department of Applied Chemistry, Yuncheng University, Yuncheng 044000, China; 2 Key Laboratory for Preparation and Application of Ordered Structural Materials of Guangdong Province, College of Chemistry and Chemical Engineering, Shantou University, Shantou 515063, China; 24rhdeng@stu.edu.cn (R.D.); 23jxxie1@stu.edu.cn (J.X.); 23jytang@stu.edu.cn (J.T.); 23wygu@stu.edu.cn (W.G.); 23zmpeng@stu.edu.cn (Z.P.)

**Keywords:** ZSM-22, Si-OH, defect healing, n-decane hydroisomerization

## Abstract

The abundant Si-OH groups serving as the defect sites in the ZSM-22 zeolite framework are not only closely associated with physicochemical properties and catalytic performance but are also the primary sites for attack by water molecules, thereby restricting applications involving or producing water. In the present study, a liquid-mediated healing process was used to convert the Si-OH groups in ZSM-22 zeolite into Si-O-Si bonds. The systematic investigation of the treatment solution’s composition and the hydrothermal conditions revealed that the crystallinity of ZSM-22 zeolite can be effectively enhanced following optimal healing treatment. Furthermore, the healed ZSM-22 zeolite exhibited enhanced ordering degree and pore connectivity without significant changes to the Si/Al and morphology. Moreover, the decline in Si-OH groups reduced the Lewis acid sites without altering the Brønsted acidity, which led to a slight decrease in the dispersion of Pt particles. In n-decane hydroisomerization, compared to the parent ZSM-22 zeolite catalyst, the healed catalyst demonstrates higher reactant conversion and increased isomer yield, attributing to its enhanced confinement within the micropore voids, which leads to a reduced yield of multi-branched isomers susceptible to cracking. This study presents a potential application of ZSM-22 zeolite in hydroisomerization for complex and challenging feedstocks.

## 1. Introduction

ZSM-22 zeolite, with one-dimensional channels (ca. 0.46~0.57 nm aperture) oriented along the [001] direction, shows great potential as a shape-selective catalyst in the oil refining and petrochemical industries, including hydroisomerization, naphtha cracking, and methanol-to-olefins reactions [1,2]. In particular, with the continuous promotion of the Fischer-Tropsch synthesis technology combined with biomass gasification and green hydrogen production to produce liquid fuels and chemicals, it is essential to significantly enhance the catalytic performance and broaden the application scenarios of ZSM-22 zeolites in the hydroisomerization reactions of long-chain linear alkanes [3,4]. A range of strategies, including heteroatom isomorphous substitution, the creation of intracrystalline mesopores, and the synthesis of nano-sized crystals, have been developed to optimize the acidity, microporous structure, and diffusion properties of ZSM-22 zeolites [5,6,7]. Consequently, the resultant ZSM-22 zeolites exhibit improved catalytic performance and altered product distribution in hydroisomerization processes. Notably, these treatment routes often result in a significant increase in Si-OH groups in the zeolite structure. For example, the desilication or dealumination employed to generate intra-crystalline mesopores usually leads to the loss of framework Si or Al atoms, thereby inevitably generating a large amount of Si-OH species. By regulating the synthesis route for the preparation of small-crystal zeolites, more terminal Si-OH groups are produced [8]. However, the Si-OH species in the zeolite structure frequently serve as the predominant sites for the adsorption of and interaction with water molecules [9,10]. Under reaction conditions characterized by elevated pressure and temperature, water molecules contribute to micropore obstruction, structural degradation, and catalyst deactivation, owing to the migration of silicon and aluminum species from the framework to the extra-framework [11]. Consequently, addressing the limitations associated with complex feedstocks containing water and producing water involves a significant role in reducing the Si-OH groups in ZSM-22 zeolite.

In comparison to the conventional synthesis route for ZSM-22 zeolites, which operate in a strongly alkaline environment, incorporating fluorine species into the initial gel offers distinct advantages. This approach not only enables the crystallization of the zeolite to occur at lower pH values, but it also significantly reduces the rates of nucleation and crystal growth. Consequently, it is anticipated that the F-containing synthesis method can produce ZSM-22 zeolites characterized by a lower presence or absence of Si-OH groups [12]. Previous reports suggested that using ionic liquids as templates and co-solvents in the fluorine-containing system could produce ZSM-22 zeolite with highly crystalline and low-defect sites [13]. However, the resultant ZSM-22 zeolites have notably large crystals exceeding 5 µm in diameter [14,15]. In the hydroisomerization of heavy linear paraffins, the Brønsted acid sites located at the micropore mouths of ZSM-22 zeolites serve as the primary sites for protonation and rearrangement following the pore mouth catalysis model [16]. The giant crystals are responsible for low catalytic efficiency due to the inaccessibility of the Brønsted acid sites located inside the one-dimensional micropores. Recently, Liu et al. developed an ionic liquid-assisted route to prepare defect-free ZSM-22 zeolite with a width diameter ranging from 50 nm to 80 nm; meanwhile, the complicated synthesis process and high cost of this zeolite significantly limit its further application [17]. Consequently, employing a post-treatment method to heal the Si-OH defects present in the ZSM-22 zeolite structure seems more practical and condonable.

Several techniques, including high-temperature treatment, silication with silane-based molecules, selective coke deposition, and metal incorporation, are available to heal the defects generated in zeolites [18,19,20]. Meanwhile, the features and catalytic performance of the healed zeolites are associated with their specific topology types and the employed healing methods. An innovative post-hydrotreatment method has recently been developed, which employs both quaternary ammonium alkali and fluoride to treat various zeolites under controlled conditions, successfully resulting in the effective transformation of Si-OH groups [21,22]. Nevertheless, the zeolites exhibit considerable differences in their framework structures and behavior. It is essential to investigate the key parameters in the liquid-mediated Si-OH healing route based on ZSM-22 zeolite and to elucidate the differences between the resulting zeolite catalyst and the parent catalyst in terms of n-decane hydroisomerization performance and product distribution.

This research systematically investigated the influence of solution composition and hydrothermal conditions on the crystallinity and structure of the ZSM-22 zeolite during liquid-mediated Si-OH healing treatment. Meanwhile, a comprehensive comparison was conducted between the healed ZSM-22 zeolite produced under optimal conditions and the parent ZSM-22 sample. As a result, the healing treatment significantly reduced the Si-OH defect sites in ZSM-22 zeolite without causing a significant change in the Si/Al ratio, morphology, and Brønsted acidity. Moreover, the healed catalyst demonstrated enhanced activity and improved yields of the desired products of n-decane hydroisomerization. This research expands the potential applications of ZSM-22 zeolite across various catalytic environments.

## 2. Results and Discussion

### 2.1. Liquid-Mediated Defect Healing Treatment

The liquid-mediated defect healing treatment of the Si-OH defect sites in the framework structure of ZSM-22 zeolite primarily involves dispersing the parent zeolite powder in an aqueous solution containing NH_4_F and TEAOH, followed by the healing treatment under hydrothermal conditions.

**Figure 1:**
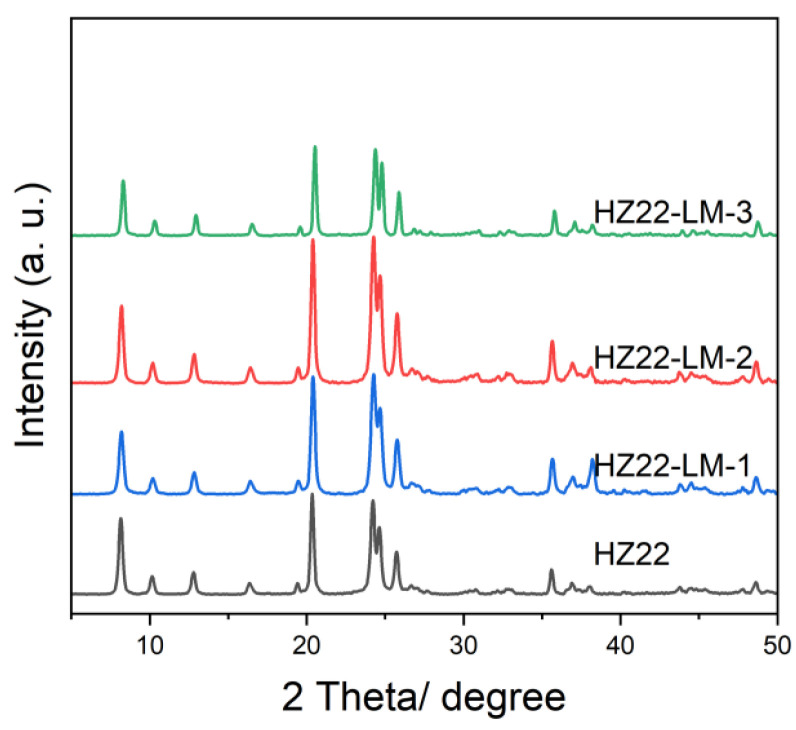
XRD patterns of the parent HZ22 and healed HZ22-LM samples prepared using different NH_4_F contents. (HZ22-LM-1: TEAOH/NH_4_F = 0.1/0.1, 120 °C, 24 h; HZ22-LM-2: TEAOH/NH_4_F = 0.1/0.4, 120 °C, 24 h; HZ22-LM-3: TEAOH/NH_4_F = 0.1/0.7, 120 °C, 24 h).

**Figure 2:**
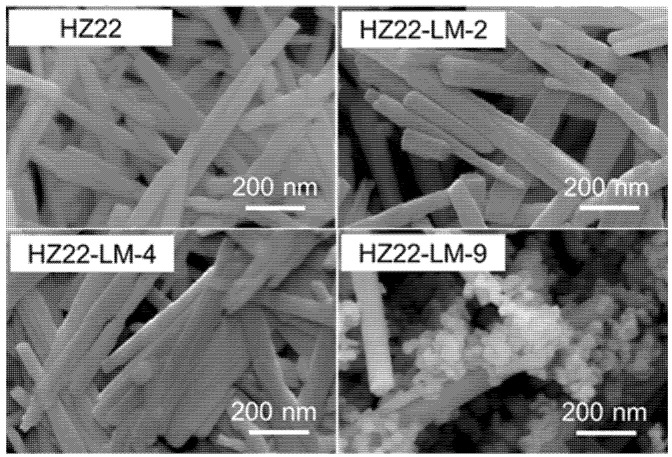
SEM images of the parent and various healed ZSM-22 zeolites (HZ22-LM-2: TEAOH/NH_4_F = 0.1/0.4, 120 °C, 24 h; HZ22-LM-4: TEAOH/NH_4_F = 0.4/0.4, 120 °C, 24 h; HZ22-LM-9: TEAOH/NH_4_F = 0.4/0.4, 150 °C, 24 h).

This research first examines the influence of varying NH_4_F concentrations in the healing solution on the relative crystallinity of as-prepared ZSM-22 zeolites. The initial molar ratio of the healing solution containing NH_4_F:TEAOH = a:0.1, where a varies from 0.1 to 0.7 (Run No. 1–3; Table 1) at 120 °C for 24 h. The XRD patterns of the parent and healed ZSM-22 samples are presented in Figure 1, and the relative crystallinities of various samples are detailed in Table 1. Compared to the parent HZ22 zeolite, the HZ22-LM-x (x = 1 and 2) samples exhibit sharper characteristic diffraction peaks attributable to TON-type zeolites, suggesting enhanced crystallinity and ordering degree. No peaks assigned to the impurities appear, indicating the absence of crystal transformation [23]. As the NH_4_F amount increases to 0.7, the relative crystallinity of the HZ22-LM-3 zeolite decreases significantly. During the liquid-mediated healing treatment process, the NH_4_F agent activates partial Si-OH groups and induces condensation between each Si-OH group. A further increase in the concentration of NH_4_F to 0.7 results in the significant etching of the ZSM-22 zeolite framework by fluoride ions, ultimately leading to structural degradation. The SEM images presented in Figure 2 indicate that both the HZ22 and HZ22-LM-2 samples have a crystal diameter of 50 to 60 nm and a length ranging from 600 to 900 nm, suggesting that the liquid-mediated healing treatment has a negligible impact on crystal morphology and size.

**Figure 3:**
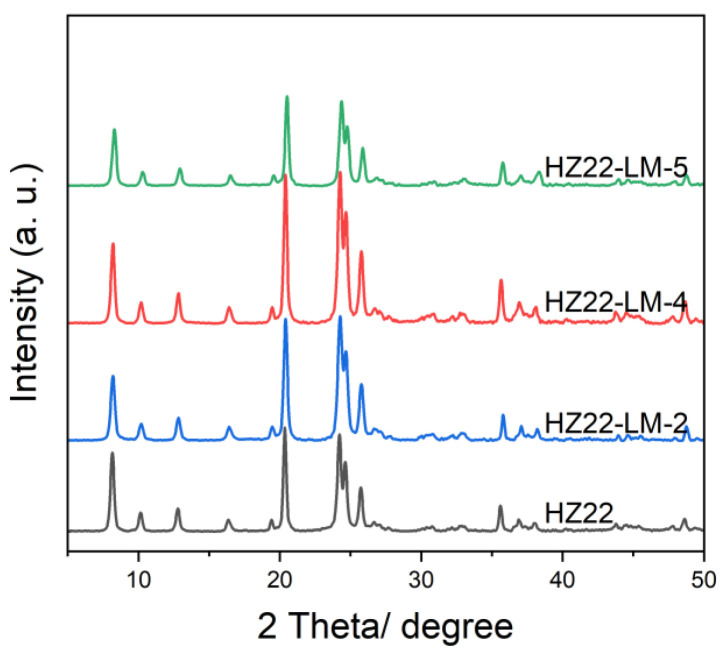
XRD patterns of the parent HZ22 and healed HZ22-LM samples prepared using different TEAOH contents. (HZ22-LM-2: TEAOH/NH_4_F = 0.1/0.4, 120 °C, 24 h; HZ22-LM-4: TEAOH/NH_4_F = 0.4/0.4, 120 °C, 24 h; HZ22-LM-5: TEAOH/NH_4_F = 0.7/0.4, 120 °C, 24 h.)

Furthermore, this work further investigates the effect of TEAOH feed amount on the crystallinity and morphology of ZSM-22 zeolites. Figure 3 shows the XRD patterns of the healed ZSM-22 zeolites obtained with an initial molar ratio of NH_4_F:TEAOH:H_2_O = 0.4:b:50, where b varies between 0.1 and 0.7 (Run No. 2, 4–5; Table 1). Consistent with the established trends regarding the influence of NH_4_F on the crystallinity of the healed samples, the crystallinity of the HZ22-LM-x samples (x = 2, 4, and 5) exhibits an initial increase as the amount of TEAOH fed into the process rises, followed by a subsequent decline. The reduction in crystallinity is similarly attributable to the structure desilication and leaching caused by the excessive alkaline TEAOH, rather than stabilizing the ZSM-22 zeolite framework. Among the healed zeolites, the HZ22-LM-4 sample, prepared with an initial ratio of NH_4_F:TEAOH:H_2_O = 0.4:0.4:50 under the hydrothermal conditions of 120 °C for 24 h, exhibits the highest relative crystallinity of 1.32. The findings indicate that the stability function of TEAOH and the activation function of NH_4_F achieve an improved equilibrium under these conditions, effectively mitigating the risk of significant framework dissolution. Additionally, the HZ22-LM-4 sample exhibits a similar crystal morphology and size, as expected.

**Figure 4:**
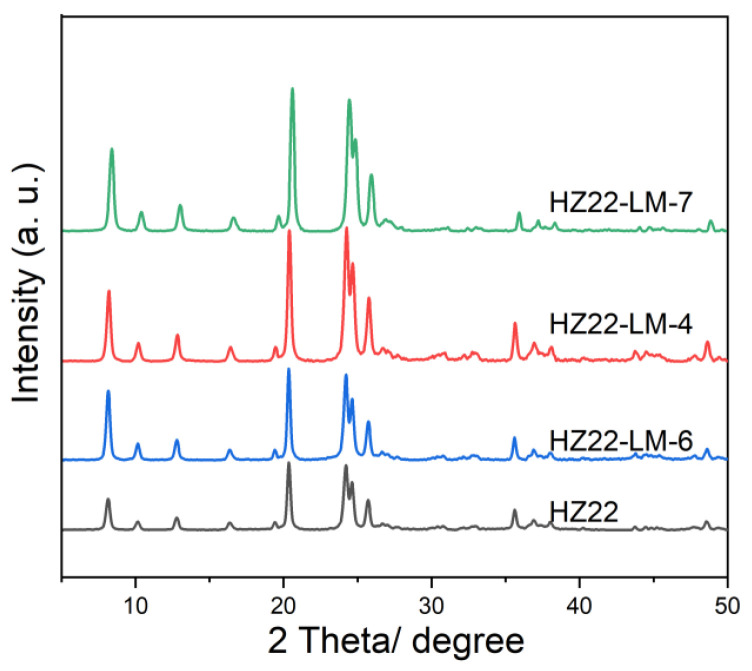
XRD patterns of the parent and healed ZSM-22 zeolite prepared under different treatment durations. (HZ22-LM-6: TEAOH/NH_4_F = 0.4/0.4, 120 °C, 12 h; HZ22-LM-4: TEAOH/NH_4_F = 0.4/0.4, 120 °C, 24 h; HZ22-LM-7: TEAOH/NH_4_F = 0.4/0.4, 120 °C, 48 h).

Based on the above healing solution ratio, a comprehensive investigation of hydrothermal conditions is conducted by systematically varying both duration and temperature. The liquid-mediated healing treatment is performed at 120 °C for durations ranging from 12 to 48 h (Run Nos. 6, 4, and 7; Table 1). As demonstrated in Figure 4 and Table 1, the crystallinity of the HZ22-LM-x samples (x = 6, 4, and 7) initially increases as the hydrothermal treatment duration is extended from 12 h to 48 h; thereafter, it stabilizes and remains unchanged. The results show that the Si-OH activation and healing process of the ZSM-22 zeolite can be completed within 24 h, and further extending the duration of hydrothermal treatment has a negligible impact on this process.

**Figure 5:**
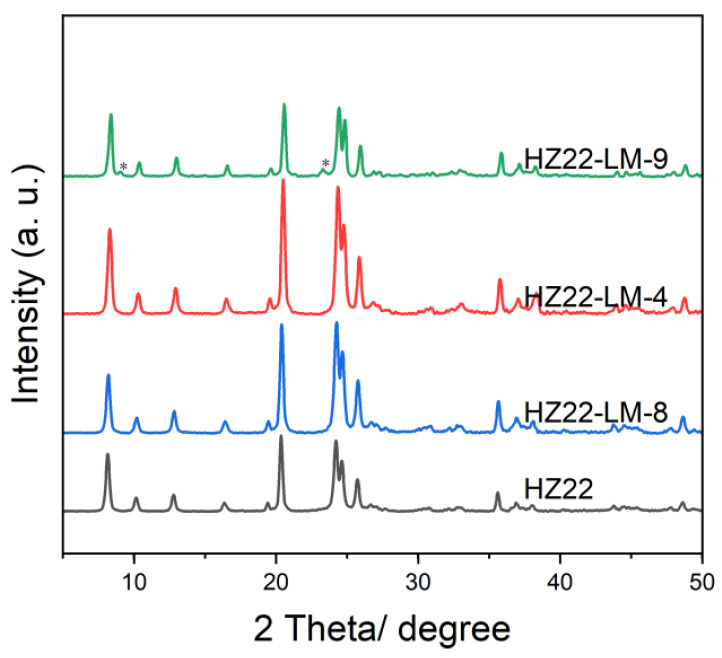
XRD patterns of the parent and healed ZSM-22 zeolite prepared under different treatment temperatures. (HZ22-LM-8: TEAOH/NH_4_F = 0.4/0.4, 100 °C, 24 h; HZ22-LM-4: TEAOH/NH_4_F = 0.4/0.4, 120 °C, 24 h; HZ22-LM-9: TEAOH/NH_4_F = 0.4/0.4, 150 °C, 24 h. The asterisks mark the diffraction peak of ZSM-5 zeolite).

Furthermore, as shown in Figure 5 and Table 1, as the treatment temperatures are set at 100 °C and 120 °C, the diffraction peak intensity and relative crystallinity of the healed samples (HZ22-LM-8 and HZ22-LM-4) are slightly different, indicating that within this range, the liquid-mediated healing treatment is insensitive to the temperature changes. However, when the treatment temperature is increased to 150 °C, diffraction peaks belonging to the ZSM-5 zeolites, which are common impurities in the synthesis of ZSM-22 [24], can be observed at 2θ = 7.9° and 23.2° in the XRD pattern of the HZ22-LM-9 sample (Run 9, Table 1). Additionally, the ZSM-5 crystals with distinct morphologies from those of ZSM-22 zeolites can be observed in the SEM image (Figure 2), indicating crystal transformation.

Based on the comprehensive systematic investigation of the compositions and hydrothermal conditions, the ratio of NH_4_F to TEAOH and the treatment temperature are identified as crucial parameters determining the healing effect of the ZSM-22 zeolite structure. Nonetheless, maintaining the appropriate parameter region remains essential in hindering the transformation of the crystal phase. Therefore, based on the relative crystallinity (Table 1) and morphology of each sample (Figure 2), it can be concluded that the optimal synthesis parameters for the HZ22-LM-4 sample are a TEAOH/NH_4_F ratio of 0.4/0.4, along with a hydrothermal treatment performed at 120 °C for 24 h.

### 2.2. Insights on the Physicochemical Properties

The HZ22-LM-4 sample, prepared under hydrothermal conditions of 120 °C for 24 h using an NH_4_F/TEAOH molar ratio of 0.4/0.4, is selected as the model material to study the changes in various crucial properties, compared to the parent HZ22 sample.

Table 2 presents the molar ratios of Si to Al in the samples before and after liquid-mediated Si-OH healing treatment. Following the healing process, the Si/Al ratio for the HZ22-LM-4 sample remains comparable to that of the parent HZ22 zeolite. This observation can be clearly explained by the fact that the transformation of Si-OH groups into Si-O-Si bonds can occur without causing significant etching of the zeolite structure due to the stability effect of the TEAOH.

**Figure 6:**
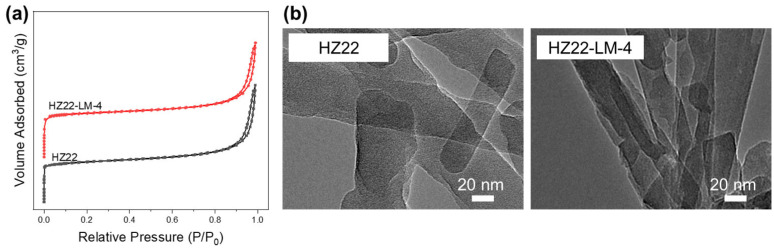
Nitrogen adsorption–desorption isotherms (**a**) and TEM images (**b**) of the parent HZ22 and healed HZ22-LM-4 samples.

Figure 6 shows the nitrogen physisorption isotherms of parent HZ22 and healed HZ22-LM-4 zeolites, and Table 2 summarizes the calculated textural features. Both samples display a typical type I physisorption isotherm (Figure 6a) with fast uptake at low relative pressure, which is characteristic of microporous zeolites [25]. The N_2_ uptake at relative pressures above 0.8, with a hysteresis loop, is attributed to the inter-crystalline mesopores formed by the random stacking of the nanocrystals. The HZ22-LM-4 sample has a higher microporous surface area (175.1 m^2^/g) and volume (0.077 cm^3^/g) compared to the parent HZ22 sample (163.7 m^2^/g and 0.071 cm^3^/g), due to the improved ordering degree of the framework resulting from the effective Si-OH healing treatment.

Similarly to the SEM observations, the HZ22 and HZ22-LM-4 samples exhibit similar needle-like morphology, with a crystal diameter ranging from 50 nm to 80 nm and a length of 700–900 nm, as shown in Figure 6b. Moreover, the well-defined lattice fringes along the (001) face illustrate the high crystallinity of the single-crystalline HZ22-LM-4 zeolite.

**Figure 7:**
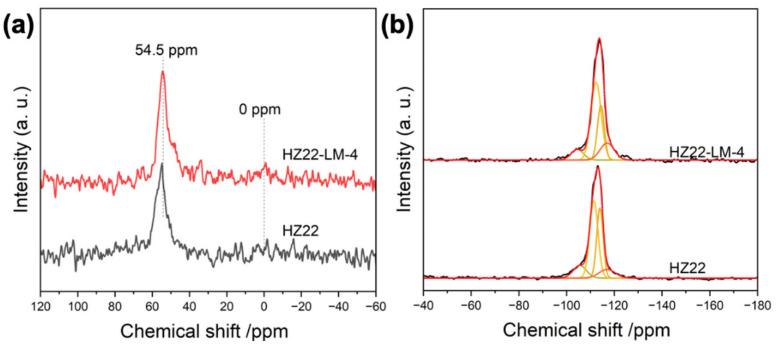
^27^Al (**a**) and ^29^Si (**b**) MAS NMR of the parent HZ22 and healed HZ22-LM-4 samples.

The ^27^Al and ^29^Si MAS NMR spectra are used to investigate the change in the coordination environment of the ZSM-22 zeolite framework before and after the healing treatment, as shown in Figure 7. The ^27^Al MAS NMR spectrum (Figure 7a) shows a dominant resonance peak at approximately 54.5 ppm, corresponding to tetrahedrally coordinated aluminum in the zeolite framework [26]. A weak resonance peak assigned to the extra-framework aluminum is observed at 0 ppm [27]. Following the defect healing treatments, the intensity of the signal at 54.5 ppm exhibits minimal variation. Due to the low concentration of aluminum species in the parent HZ22 zeolite, distinguishing the effects of the healing treatment on the extra-framework aluminum from the relatively high background noise presents a significant challenge. The ^29^Si MAS NMR spectra presented in Figure 7b display multiple signals in the range of −100 ppm to −120 ppm, which correspond to the various coordination structures of the Si species. The absence of the peak at −91 ppm indicates that the germinal Q2 Si species ((HO)_2_-Si-(OSi)_2_) are negligible in all high-silica zeolites. The observed strong signals, ranging from −108 to −120 ppm, indicate the dominance of the Q4 (Si-(OSi)_4_) species [28]. Generally, the signal observed within the range of −100 ppm to −108 ppm is attributed to the overlapping contributions from the silicon species of (HO)-Si-(OSi)_3_ and (AlO)-Si-(OSi)_3_ [29]. Given the comparable Si/Al ratios and the aluminum coordination environment present in both samples (Table 2, Figure 7a), the pronounced reduction in the Q3 signal of the HZ22-LM sample indicates that the healing treatment effectively removes the Si-OH groups. The contribution of the Q3 signal to the total spectral intensity is 6.1% in the HZ22-LM-4 sample, whereas in the HZ22 zeolite, this contribution is 12.1%. Furthermore, the liquid-mediated treatment improves the structural ordering of zeolites, as demonstrated by the increased intensity of the Q4 signals. Combined with the above characterizations, this liquid-mediated treatment is an effective method for enhancing the ordering degree of the ZSM-22 zeolite structure and improving connectivity levels without altering the Si/Al ratio or crystal morphology.

**Figure 8:**
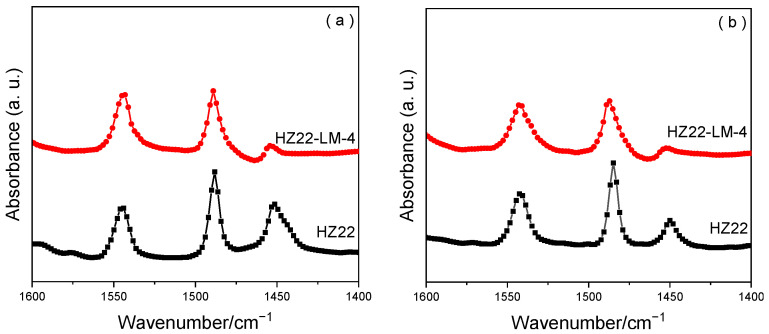
Py-FTIR spectra of the HZ22 and HZ22-LM-4 zeolites after degassing at 200 °C (**a**) and 350 °C (**b**).

The pyridine FT-IR measurements are performed for the parent HZ22 zeolite and the healed HZ22-LM-4 sample in Figure 8 and Table 2. Three distinct vibration peaks can be observed in the relevant region, spanning from 1400 cm^−1^ to 1600 cm^−1^. The peak at 1544 cm^−1^ is associated with the formation of pyridinium ions on Brønsted acid sites, while the peak at 1455 cm^−1^ is linked to the coordination of pyridine molecules to Lewis acid sites, and the peak at 1490 cm^−1^ is assigned to both Lewis and Brønsted acid sites [30].

As present in Table 2, the HZ22 and HZ22-LM-4 samples exhibit similar density and distribution of the Brønsted acid sites. Notably, the Si-OH healing treatment markedly alters the Lewis acidity of these samples. Specifically, compared to the parent HZ22 zeolite, the Lewis acidity of the HZ22-LM-4 sample exhibits a noticeable reduction, particularly in the detection of strong Lewis acid sites (350 °C), which becomes challenging. The Si-OH groups, always serving as the defect sites in the lattice framework, are closely associated with the Lewis acid sites, due to the electronically unsaturated state of the Si atoms bonding with three lattice oxygen atoms and one hydroxyl group [31]. The liquid-mediated healing treatment leads to the formation of Si-O-Si bonds with full coordination, thereby resulting in the decline of Lewis acid sites.

In n-paraffin hydroisomerization, Pt metal sites are responsible for the (de)hydrogenation function, playing a crucial role in affecting catalytic activity and stability. In this study, H_2_PtCl_6_·(H_2_O)_6_ is employed as a platinum precursor to deposit 0.5 wt% of Pt onto the zeolite supports, thereby obtaining the bifunctional catalyst, as described in the Experimental section.

It is essential to investigate the dispersion of the Pt particles and the ratio of metal sites to acid sites, as this factor influences the mechanisms of hydroisomerization and hydrocracking reactions, as well as the resulting product distribution [32]. As outlined in Table 3, the Pt particle diameters for the Pt/HZ22 and Pt/HZ22-LM-4 catalysts consistently exceed 2.0 nm, making them difficult to locate within the zeolite micropores. This observation suggests that the majority of the Pt particles are randomly distributed at the entrances of the micropores and the external surfaces of these ZSM-22 crystals. When comparing the Pt/HZ22 catalyst with the Pt/HZ22-LM-4 catalyst, it is evident that the latter exhibits a reduction in Pt dispersion and an increase in Pt particle diameter. This finding indicates that the transformation of Si-OH into Si-O-Si groups through the liquid-mediated healing method diminishes the interaction between the support and the metal Pt. As a result, a reduction in platinum dispersion, accompanied by an increase in the particle size, is observed over the Pt/HZ22-LM-4 catalyst.

This phenomenon can be elucidated by the interaction between the Pt metal and the Si-OH groups of the zeolites, facilitated through the formation of Si-O-OPt bonds [33,34]. Therefore, the reduction in Si-OH groups is attributable to the aggregation of Pt particles observed on the Pt/HZ22-LM-4 catalyst.

Regarding the bifunctional catalysts employed in n-alkane hydroisomerization, the n_Pt_/n_A_ ratio is regarded as a crucial indicator for determining the course of the reaction process. The calculation of n_Pt_ is predicated upon the metal Pt particle size determined by CO chemisorption, in conjunction with the molar mass and loading content of Pt. The concentration of Brønsted acid sites, assessed through pyridine-adsorbed FTIR, is utilized as the n_A_ value. As listed in Table 3, all catalysts exhibit an n_Pt_/n_A_ ratio exceeding 0.17, irrespective of the variations in Pt dispersions and Brønsted acidity. G. Giannetto et al. demonstrated that the n_Pt_/n_A_ ratio strongly affected the activity and formation order of iso-products. When the value of n_Pt_/n_A_ exceeds 0.17, the transformation of n-alkane proceeds via a sequential process in which mono-branched, multi-branched, and cracked products are generated successively. Each olefin intermediate interacts with a limited number of acid sites located between hydrogenation sites and experiences only a single transformation, either branching or cracking. Furthermore, the hydroisomerization reaction functions as the rate-limiting step within the acid-catalysis process [35]. The catalytic performance and product distribution of individual catalysts in the hydroisomerization of n-decane are primarily influenced by the differences in the zeolite supports utilized.

### 2.3. Catalytic Performance in N-Decane Hydrosomerization

**Figure 9:**
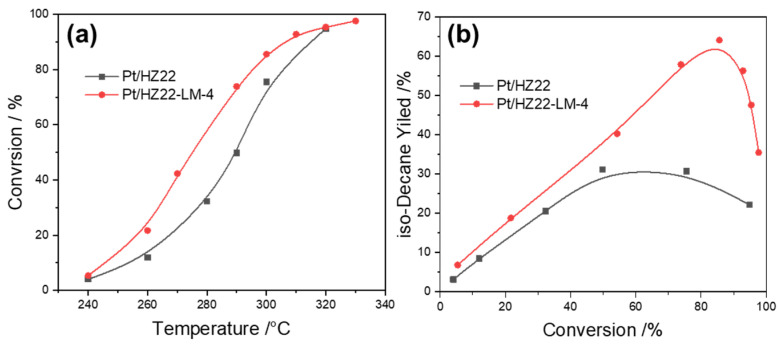
Activity and yield plots for n-decane hydroisomerization for the Pt/HZ22 (black lines) and Si-OH-healed Pt/HZ22-LM-4 catalysts (red lines). n-Decane conversion versus reaction temperature (**a**) and isomer yield versus conversion (**b**). (Detailed reaction conditions are provided in the Catalytic Evaluation Section).

The investigation into the effects of the liquid-mediated Si-OH healing process on catalytic activity and isomer yield focuses on the hydroisomerization of n-decane. The results, illustrated in Figure 9, indicate that this healing treatment significantly enhances the activity of the Pt/HZ22-LM-4 catalyst in comparison to the parent Pt/HZ22 catalyst. Table 2 and Table 3 reveal comparable Si/Al ratios, Brønsted acidity, microporous surface area, and n_Pt_/n_A_ ratios exceeding 0.17 for both typical catalysts. Therefore, the observed differences in catalytic activity can be attributed to the framework lattice structure of the ZSM-22 zeolites. This healing treatment results in an elevated ordering degree of the microporous structure, thereby creating a more constrained environment. The confinement of the reactant molecule, the associated adsorption enthalpy, and the entropy of the system are linked to the spatially defined local reaction environments present within the zeolite channel [36,37]. These factors collectively influence the overall reaction dynamics and efficiency [38]. Consequently, the enhanced micropore confined environment resulting from the healing of Si-OH groups in the ZSM-22 framework facilitates the altered stabilization of the key carbenium ion intermediates, significantly influencing the catalytic activity observed in the hydroisomerization of n-decane.

Figure 9b illustrates that as the conversion of n-decane increases, the isomer yield across both catalysts initially increases; however, it subsequently declines with further increases in reaction temperature. This decreasing trend is attributable to the highly endothermic feature of the hydrocracking reaction. Compared to the Pt/HZ22 catalyst, the Pt/HZ22-LM-4 catalyst exhibits a significantly enhanced isomer yield, underscoring the effectiveness of reducing Si-OH defect sites in improving catalytic performance during n-decane hydroisomerization. The highest isomer yield recorded is 64%, achieved with the Pt/HZ22-LM-4 catalyst. In contrast, the maximum isomer yield obtained with the Pt/HZ22 catalyst is only 31%, while the yield to cracked products with a carbon chain length lower than ten reaches 69%.

**Figure 10:**
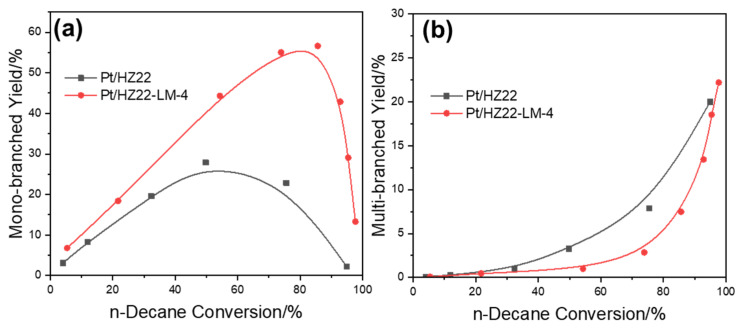
Yield of isomerized products ((**a**) mono-branched iso-decanes, (**b**) multi-branched iso-decanes) for the Pt/HZ22 (black lines) and Si-OH healed Pt/HZ22-LM-4 catalysts (red lines) (Reaction conditions are provided in the Catalytic Evaluation Section).

To elucidate the factors contributing to the differing hydroisomerization performances between the Pt/HZ22 and Pt/HZ22-LM-4 catalysts, which possess comparable Si/Al ratios and Brønsted acidity, the iso-products are classified into mono-(methylnonane) and multi-branched isomers (dimethyloctane and trimethylheptane), as depicted in Figure 10. Based on the classical and “ideal” bifunctional catalysis mechanism [34], the n-decane reactant is transformed successively into both mono- and multi-branched isomers when employing the bifunctional catalysts with an n_Pt_/n_A_ ratio exceeding 0.17. Therefore, the yield of multi-branched isomers sharply increases under high reaction temperatures. Notably, at comparable conversion levels, the yield of mono-branched isomers produced by the Pt/HZ22-LM-4 catalyst exceeds that of the Pt/HZ22 catalyst. Conversely, the yield of multi-branched isomers demonstrates a markedly different trend across both catalysts.

As is well understood, during the n-paraffin hydroisomerization over the ZSM-22 zeolite catalyst, the formation and subsequent transformation of alkyl carbenium ions, acting as pivotal reaction intermediates via branched carbon skeletons, occur at the micropore mouths [38]. Significantly, these alkyl carbenium ions are transient, highly energetic, and positively charged species. Their stability is improved through interactions with basic oxygen atoms within the zeolite lattice. This effect is more significant in the high-ordering degree lattices, attributable to the enhanced electric field and electric field gradient [39].

However, the Si-OH groups, serving as defect sites in the ZSM-22 lattice, are accountable for the less confined environment and exhibit reduced efficiency in stabilizing alkyl carbenium ions. This leads to the further rearrangement of the alkyl carbenium ions and the formation of di- and tri-branched carbon skeletons, which are susceptible to cracking due to multiple β-scission modes. Thus, transforming the Si-OH groups in the parent HZ22 zeolite into saturated Si-O-Si bonds in the HZ22-LM-4 sample, which hinders the further rearrangement of alkyl carbenium ions and the formation of multi-branched isomers. Therefore, this limitation from the strict micropore confinement environment of the healed catalyst (Pt/HZ22-LM-4) contributes positively to enhancing the overall isomer yield.

**Figure 11:**
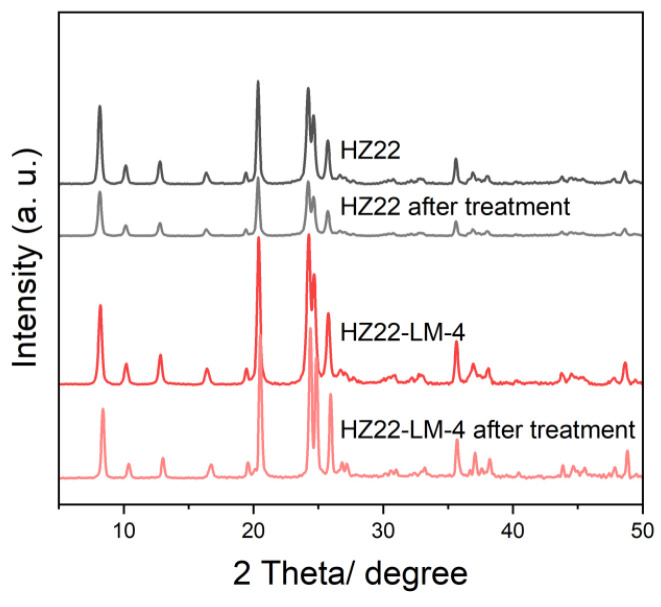
XRD patterns of the HZ22 and HZ22-LM-4 samples before and after the hydrothermal treatment under 160 °C for 48 h in the autoclave.

It is widely recognized that the Si-OH groups in the zeolite framework serve as the primary sites for adsorption and attack of the water molecules, leading to the corrosion and degradation of the aluminosilicate structure. The HZ22 and HZ22-LM-4 samples are subjected to a hydrothermal environment to investigate the effect of Si-OH defect healing on water erosion resistance. Following hydrothermal treatment of the parent HZ22 sample at 160 °C for 48 h under continuous stirring, a significant reduction in the intensity of the diffraction peaks is observed, as illustrated in Figure 11.

The relative crystallinity of the parent HZ22 zeolite exhibits a significant decline from 1.00 to 0.25, which can be attributed to structural degradation resulting from water etching. In contrast, the HZ22-LM-4 sample demonstrates an almost unchanged relative crystallinity, exhibiting a decrease of only 0.05. This finding suggests that the Si-OH-healed HZ22-LM-4 sample exhibits good hydrothermal stability under these challenging conditions.

**Figure 12:**
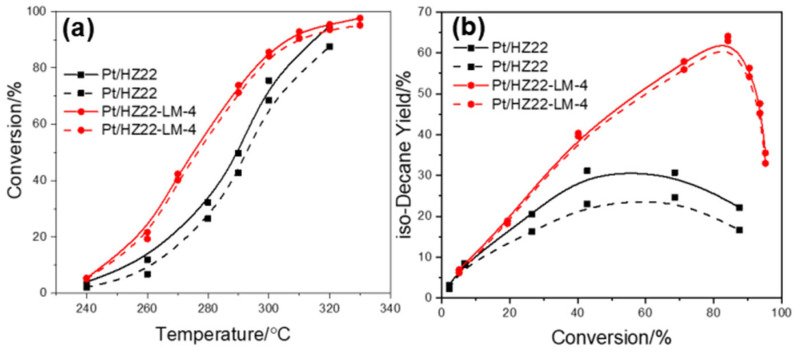
Hydroisomerization performance over the Pt/HZ22 and Pt/HZ22-LM-4 catalysts without (solid line) and with (dashed line) n-butanol in the feedstock. n-Decane conversion versus reaction temperature (**a**) and isomer yield versus conversion (**b**). (Reaction conditions: 240 °C~330 °C, 2.0 MPa, LHSV = 2.0 h^−1^, H_2_/n-decane volume ratio = 600).

Moreover, to assess the resistance of these catalysts against oxygen-containing compounds in the hydroisomerization of n-decane, 0.5 wt% of n-butanol is introduced into n-decane as the feed under the same reaction conditions. As shown in Figure 12a, the model n-butanol results in a notable reduction in the n-decane conversion of the parent Pt/HZ22 catalyst, while exerting minimal influence on the activity of the Pt/Z22-LM-4 catalyst. This phenomenon is attributed to n-butanol competing with n-decane for adsorption on the active sites of the metal Pt and zeolite during hydroisomerization. Furthermore, the effect of n-butanol on the isomer yields of these catalysts was markedly different. For the parent Pt/HZ22 catalyst, the influence of n-butanol on the reduction of isomer yield is apparent, with the maximum isomer yield decreasing from 31.1% to 24.6%, as shown in Figure 12b. The decrease in iso-decane yield over the Pt/HZ22-LM-4 catalyst is not apparent. The maximum isomer yield decreases by less than 2%, from 64.1% to 63.0%, indicating the enhanced resistance of this catalyst to oxygen-containing compounds relative to the parent catalyst.

## 3. Materials and Methods

### 3.1. Chemicals and Reagents

The tetraethylammonium hydroxide (TEAOH, 25 wt% in water) was purchased from Aladdin in Shanghai, China. The ammonium fluoride (NH_4_F, 99.5%) was obtained from Macklin. To prepare the bifunctional catalyst, the chloroplatinic acid hexahydrate (H_2_PtCl_6_·6H_2_O, Pt ≥ 37 wt%) was used as the precursor of the Pt metal. The hydroisomerization performance was evaluated using n-decane (n-C_10_, Aladdin) as the feed reactant. All of these chemicals were used as received, without any additional purification.

### 3.2. Zeolite Synthesis

The ZSM-22 zeolites were well-prepared according to the recipe described in the literature [39] using 1,6-diaminohexane as the organic structure directing agent (OSDA). The initial molar composition of the gel was 1.0 SiO_2_: 0.13 K_2_O: 0.0125 Al_2_O_3_: 0.3 OSDA: 40 H_2_O. After hydrothermal crystallization at 160 °C for 48 h, the solid material was filtered, washed to a neutral pH, dried overnight at 120 °C, and then calcined at 550 °C for 8 h. The calcined zeolites (denoted as Z22) underwent a third ion exchange process in an aqueous 0.5 M NH_4_Cl solution at 80 °C for 6 h to produce NH_4_^+^ samples identified as NH_4_-Z22. Subsequently, the NH_4_-Z22 samples were subjected to calcination at 550 °C for 4 h, resulting in the formation of protonic zeolites designated as HZ22.

### 3.3. Si-OH Defect Healing Treatment

The Z22 sample underwent liquid-mediated defect healing treatment by being suspended in a post-treatment solution containing NH_4_F and TEAOH with different molar compositions of TEAOH:NH_4_F:H_2_O = (0.1~0.7):(0.1~0.7):50. The weight ratio of the powdered zeolite to the solution was set at 1:30. The suspension was stirred at room temperature for 0.5 h and then transferred to a Teflon-lined autoclave for a hydrothermal treatment between 100 °C and 170 °C for various durations (from 12 to 72 h) under static conditions. After the treatment, the solid products were collected by vacuum filtration and washed with deionized water until the pH was neutral. Finally, the treated samples were dried, calcined, and ion-exchanged to obtain the protonic zeolite sample denoted by HZ22-LM-x, where x represents the different treatment conditions. The detailed compositions and hydrothermal process for the defect healing of protonic ZSM-22 zeolites are collected in Table 1.

### 3.4. Catalyst Preparation

To obtain the bifunctional catalyst for the catalytic assessment, the parent zeolite and typically healed zeolite (HZ22-LM-4) were extruded and subsequently sieved to achieve a particle size of 20~40 mesh. Before the impregnation process, the zeolite particles were transferred to a 50 mL two-neck flask and subjected to vacuum drying overnight. The H_2_PtCl_6_·6H_2_O was first dissolved in deionized water to prepare an impregnation solution of the Pt precursor with a Pt concentration of 1.0 wt%. While the zeolite sample remained under vacuum within the flask, the Pt precursor solution was added dropwise to the zeolite support to develop a bifunctional catalyst with a Pt loading of 0.5 wt%. Then, the impregnated zeolite was dried under static vacuum at room temperature for 1 h, followed by 6 h in the vacuum oven at 60 °C and overnight at 120 °C. The calcination process was conducted at 400 °C for 4 h with a heating rate of 1 °C/min. Based on the zeolite supports utilized, the resulting bifunctional catalysts were denoted as Pt/HZ22 and Pt/HZ22-LM-4.

### 3.5. Characterizations

The X-ray diffraction (XRD) patterns were measured by the Rigaku Smartlab X-Ray diffractometer with a Cu Kα radiation source (40 kV, 30 mA). The measurements used a step size of 0.05° within the range of 2θ = 5° to 50°. The relative crystallinities of various zeolites are calculated based on the integration of the peaks at 2θ = 8.1°, 20.3°, 24.1°, and 25.6°, assuming a crystallinity of 1.00 for the parent HZ-22 zeolite. Morphology and crystal size were characterized using a field emission scanning electron microscope (SEM, GeminiSEM 300, Oberkochen, Germany) and transmission electron microscopy (TEM, Thermo Scientific Talos F200X, Waltham, MA, USA) with an accelerating voltage of 200 kV. The Si/Al molar ratio was determined using an X-ray fluorescence spectrometer (XRF, Rigaku ZSX Primus IV, Tokyo, Japan). N_2_ physisorption isotherms at −196 °C were obtained using a Micromeritics ASAP 2460 analyzer to assess the porosity characteristics of zeolites. Before measurements, the zeolites were degassed at 350 °C for 8 h to eliminate the adsorbed moisture and impurities. The Brunauer–Emmett–Teller (BET) and t-plot methods were used to calculate the specific surface area and micropore volume.

^29^Si magic-angle spinning nuclear magnetic resonance (^29^Si MAS NMR) spectra were recorded on a Bruker Avance III 600 MHz spectrometer at 119.2 MHz with a spinning speed of 12 kHz. The chemical shift was referenced to kaolin at −91.5 ppm. The intensity of the ^29^Si NMR results was normalized by dividing the raw intensity by the weight, and the spectra were deconvoluted using the Gaussian Lorentz model.

The pyridine Fourier-transformed infrared (Py-IR) spectra of the zeolites were obtained using a Nicolet 410 FTIR spectrometer within the 400~4000 cm^−1^ range. The raw materials were compacted into self-supported thin pellets before analysis. Subsequently, the pellets were introduced into the FTIR cell, which was then connected to a vacuum line. The samples were subjected to a heating process at 400 °C for 1.0 h under a pressure of 10^−6^ Torr, followed by spectral acquisition at 30 °C, 200 °C, and 350 °C after the injection of Pyridine gas for 0.5 h at room temperature. The resulting spectra were subsequently normalized based on the weight of the self-supported disks.

The CO-chemisorption was performed to assess the dispersion and size of the Pt particles on the bifunctional catalysts using the Micromeritics ASAP 2920 analyzer from the United States (Norcross, GA, USA). The fresh catalyst was in situ reduced in the H_2_ flow (50 mL/min) at 350 °C for 2 h. The reduced catalyst was improved to 400 °C under a He atmosphere to remove the adsorbed H_2_ molecules. After cooling to 50 °C, pulses of 5% CO/95% He were injected until adsorption saturation was achieved. The average diameter of Pt particles was estimated from the Pt dispersion (CO/Pt) using the equation D (nm) = 1.13/(CO/Pt).

### 3.6. Catalytic Evaluation

n-Decane (n-C_10_) was employed as the model reactant to evaluate the hydroisomerization performance of the representative catalysts. The catalytic assessments were performed in a high-pressure fixed-bed reactor. Following the packing of the catalyst (1.5 mL) into the reaction tube, the reduction process was carried out in a H_2_ atmosphere with a GHSV of 1200 h^−1^ at 350 °C for 4.0 h under 0.2 MPa. Following cooling to the initial reaction temperature, the pressure was adjusted to 2.0 MPa, and the reactant was then injected using the pump, employing an H_2_/n-decane volume ratio of 600 and a liquid LHSV value of 2 h^−1^. The reaction temperature was increased incrementally, and products were collected for two hours at each test point. The reaction effluent was separated and collected utilizing a cold trap maintained at 5 °C. An online gas chromatograph (GC, Agilent 7820A, Santa Clara, CA, USA) was used to analyze the gaseous products, which predominantly comprised C_1_–C_7_ hydrocarbons. The liquid products were analyzed using an offline gas chromatograph (Agilent 8860) with a Flame Ionization Detector (FID) and a PONA capillary column. The carbon balances were maintained at over 98% by calculating the liquid and gas products.

## 4. Conclusions

As the complexity of the reactants involved in linear paraffin hydroisomerization increases and the requirements of application scenarios become more stringent, regulating ZSM-22 zeolite to achieve higher isomer yields may not be sufficient to address all operational needs. Improving the water resistance of ZSM-22 zeolites is crucial, especially with the increasing use of biomass reactants on a large scale. Organic templates and alkaline synthesis conditions contribute to the abundant presence of Si-OH groups in the structure of aluminosilicate ZSM-22 zeolites. Moreover, the Si-OH is closely linked to the acidity and shape selectivity of the zeolite and serves as the primary site for water attack. In this study, a liquid-mediated treatment approach was employed to heal the Si-OH bonds, facilitating their transformation into Si-O-Si groups by synergistically combining the activation properties of NH_4_F with the stabilizing effects of TEAOH. This work conducted a systematic investigation of the initial compositions and hydrothermal parameters. The balance between NH_4_F and TEAOH is essential for effectively remedying Si-OH defect sites and preventing excessive etching of ZSM-22 zeolites. Moreover, this route is insensitive to the duration of the hydrothermal treatment, but high temperatures can readily transform the ZSM-22 crystals into impurities. Compared with the parent ZSM-22 zeolite, the healed zeolite produced under optimal healing conditions demonstrates enhanced crystallinity, increased microporous surface area, and a more significant proportion of tetra-coordinated silicon species, which are accompanied by a considerable reduction in Si-OH groups and Lewis acidity. In the hydroisomerization of n-decane, the confined environment of the ZSM-22 zeolite micropores, facilitated by healing Si-OH defects, results in enhanced catalytic activity and isomer yield. This improvement arises from the limitation imposed on the formation of easily cracked multi-branched isomers due to the restricted spatial conditions. Moreover, the comparison of the hydrothermal stability of ZSM-22 zeolites before and after the healing treatment indicates that the liquid-mediated treatment effectively enhances the resistance of ZSM-22 zeolite to extreme hydrothermal conditions. Research on the healing of Si-OH sites in zeolite crystals through an effective post-treatment method provides insights into ZSM-22 zeolites and hydroisomerization catalysts in systems that contain or generate water.

## Figures and Tables

**Table 1:** Liquid-mediated healing treatment parameters and characteristics of the ZSM-22 zeolites.

Run	Samples	Compositions	Conditions		Phases ^a^	Relative Crys. ^b^
		TEAOH/NH_4_F mol/mol	Temp./°C	Dura./Hours		
	HZ22	--			TON	1.00
1	HZ22-LM-1	0.1/0.1	120	24	TON	1.08
2	HZ22-LM-2	0.1/0.4	120	24	TON	1.19
3	HZ22-LM-3	0.1/0.7	120	24	TON	0.83
4	HZ22-LM-4	0.4/0.4	120	24	TON	1.32
5	HZ22-LM-5	0.7/0.4	120	24	TON	0.93
6	HZ22-LM-6	0.4/0.4	120	12	TON	1.06
7	HZ22-LM-7	0.4/0.4	120	48	TON	1.35
8	HZ22-LM-8	0.4/0.4	100	24	TON	1.13
9	HZ22-LM-9	0.4/0.4	150	24	TON + MFI	--

^a^ Determined by the XRD technique; ^b^ the crystallinity of the parent HZ22 sample is defined as 1.00.

**Table 2:** Characteristics of the parent HZ22 and healed HZ22-LM-4 samples.

Samples	Si/Al Ratios ^a^	S_Micro_ m^2^/g	V_Micro_ cm^3^/g	Acidity (µmol/g) ^b^			
				Brønsted		Lewis	
				200 °C	350 °C	200 °C	350 °C
HZ22	38.1	163.7	0.071	116.2	80.1	23.6	22.3
HZ22-LM-4	37.0	175.1	0.077	118.7	85.4	7.8	3.9

^a^ Determined by ICP-OES measurement. ^b^ Obtained from the Pyridine-adsorbed FTIR results.

**Table 3:** Properties of the metal Pt on the reduced catalysts.

Catalysts	d CO-Chem ^a^ (nm)	Dispersion ^a^ (%)	n_Pt_/n_A_ ^b^ (mol/mol)
Pt/HZ22	2.4	46.2	0.25
Pt/HZ22-LM-4	2.8	32.4	0.21

^a^ Average diameter and dispersion of the metal Pt particles determined by CO-chemisorption. ^b^ Calculation based on the Pt sites determined by CO-chemisorption and Brønsted acid sites quantified by pyridine FT-IR.

## Data Availability

Data of this study are available within the article and from the corresponding authors on reasonable request.
